# Individual participant data (IPD)-level meta-analysis of randomised controlled trials among dark-skinned populations to estimate the dietary requirement for vitamin D

**DOI:** 10.1186/s13643-019-1032-6

**Published:** 2019-05-28

**Authors:** Kevin D. Cashman, Christian Ritz

**Affiliations:** 1 Cork Centre for Vitamin D and Nutrition Research, School of Food and Nutritional Sciences, University College Cork, Cork, Ireland; 2 Department of Medicine, University College Cork, Frederiksberg C, Ireland; 3 Department of Nutrition, Exercise and Sports, Faculty of Science, University of Copenhagen, Frederiksberg C, Denmark

**Keywords:** Vitamin D requirements, Ethnic-related differences, Individual participant data-level meta-regression analyses, Dietary Reference Values, RCT

## Abstract

**Background:**

Estimation of the dietary requirements for vitamin D is crucial from a public health perspective in providing a framework for the prevention of vitamin D deficiency. It has been shown that pooling individual participant-level data (IPD) from selected randomised controlled trials (RCTs) of white children and adults facilitated the generation of more accurate estimates of the vitamin D requirement. Recent RCT data suggest the vitamin D requirement of dark-skinned, particularly black, individuals, an at-risk group of vitamin D deficiency, is greater than those of white counterparts. Thus, we wished to develop a study protocol for the conduct of an IPD-level meta-analysis of vitamin D requirements using data from appropriate vitamin D RCTs in dark-skinned population subgroups.

**Methods:**

The study protocol details the steps needed within such an IPD meta-analysis which will include its registration, constituent systematic review to identify all appropriate RCTs on the basis of pre-specified eligibility criteria, the associated data collection, handling, and synthesis, as well as checking the integrity of the IPD, followed by implementation of a one/two-stage IPD meta-analysis and derivation of vitamin D requirement estimates.

**Discussion:**

As dark-skinned population subgroups are at increased risk of vitamin D deficiency, further investigation of dietary recommendations for vitamin D in these subgroups is needed. We strongly believe that application of an IPD-based meta-analysis is a highly strategic approach by which to undertake some of this further investigation. Such IPD-based analysis, however, will need collaboration across the principal investigators of the identified RCTs meeting with the eligibility criteria, and the availability of this study protocol will be important to highlight the potential of IPD-based analysis for estimation of the dietary requirement for vitamin D for this particular population subgroup as well as for other at-risk target populations.

**Systematic review registration:**

PROSPERO International Prospective Register of Systematic Reviews (registration number: CRD42018092343).

**Electronic supplementary material:**

The online version of this article (10.1186/s13643-019-1032-6) contains supplementary material, which is available to authorized users.

## Background

### Introduction

Vitamin D deficiency has significant implications for human health throughout life and impacts upon healthy growth and development and successful ageing [1]. In North America and Europe, African-American and other dark-skinned racial/ethnic subgroups are at a much higher risk of vitamin D deficiency compared to their white counterparts. For example, the prevalence of serum 25-hydroxyvitamin D (25(OH)D) < 30 nmol/L (a threshold used by several authorities to represent increased risk of vitamin D deficiency [2–5]) in white, Hispanic, and African-American individuals in the USA is 2.3%, 6.4%, and 24%, respectively [6]. Likewise in Europe, recent reports show that dark-skinned racial/ethnic subgroups in Finland, Norway, and the UK have much higher (3- to 71-fold) prevalence of serum 25(OH)D < 30 nmol/L than white populations [7, 8].

Dietary Reference Values (DRVs) for vitamin D are estimates of the dietary requirements for the vitamin. They are estimated as vitamin D intakes that will maintain serum 25(OH)D levels of either half (50%) or nearly all (97.5%) of individuals over a predefined threshold; they are found through inverse regression applied after having fitted suitable linear or nonlinear regression models that describe the dose-response relationship between vitamin D intake and serum 25(OH)D [9, 10]. DRVs are crucial from a public health perspective in providing a framework for the prevention of vitamin D deficiency and optimising vitamin D status of individuals [11]. Despite the fact that DRVs for vitamin D have been re-evaluated by several authorities in the past 7 years (see [12, 13], for reviews), in most cases, these recommendations were established for the entire population and not delineated by ethnic subgrouping. Such recommendations were based at the time on an assumption that the requirements between white and other racial groups do not differ, largely due to the absence of data; an area identified as a key knowledge gap by a number of authorities [2, 3, 14].

Recent data from a number of randomised controlled trials (RCTs) that compared white versus black and other dark-skinned population subgroups [15–17] suggest this assumption may not be correct, which, in turn, may mean the established dietary requirements for vitamin D will not provide the level of population protection expected for these dark-skinned population subgroups with consequences for the prevention of vitamin D deficiency. There have been several RCTs of black (African-American/East African descent) women and dark-skinned children (i.e. those with Fitzpatrick skin types V and VI) aimed at establishing the dietary requirement for vitamin D, but for the most part, the estimates have been quite variable (20–52 μg/d, using the 50 nmol/L threshold of serum 25(OH)D) [15–22].

Instead of relying on data from one specific study undertaken in a particular context, it has been suggested that by considering data from several studies ensures greater representativity [3]. This approach has been employed by a number of authorities who re-evaluated their DRV for vitamin D [2–4], using a standard meta-regression approach that relied on aggregate (group mean) data. Based on the fitted meta-analysis model, DRVs were derived using inverse regression. Recently, it has, however, been suggested that an individual participant data (IPD) meta-analysis would allow for the estimation of more appropriate DRVs [23, 24]. Specifically, the IPD meta-analysis reported by us recently [23] resulted in estimated DRVs that were considerably different from those of the agencies that used the standard meta-analysis approach.

### Objective

The aim of this work is to describe a study protocol detailing the steps for carrying out IPD meta-analysis of randomised controlled trials of vitamin D supplementation in order to estimate the dietary requirement for vitamin D for dark-skinned individuals.

## Methods/design

### Adherence to IPD guidelines

The current protocol follows the general guidance provided recently as part of the PRISMA (Preferred Reporting Items for Systematic Reviews and Meta-Analyses)-IPD statement [25]. In particular, the PRISMA-IPD method-related topics, numbered 5 to 16 within the statement’s checklist, have been considered carefully and integrated into the development of the current protocol. The overall process can be considered a set of sequential steps starting with a systematic review to identify the appropriate vitamin D RCTs and terminating in statistical analyses that estimate the dietary requirement for vitamin D in dark-skinned individuals, utilising the pooled data from all included RCTs.

### Registration

The IPD meta-analysis, which is described in this study protocol, was registered with the PROSPERO International Prospective Register of Systematic Reviews (registration number: CRD42018092343; http://www.crd.york.ac.uk/PROSPERO/display_record.php?ID=CRD42018092343 RecordID = 92343).

### Ethics approval

Approval by a research ethics committee to conduct this meta-analysis will not be required because it involves statistical analyses of de-identified data that have already been collected for a separate purpose.

### Systematic review to identify eligible papers

#### Eligibility criteria

The eligibility criteria used are closely aligned with the ones used in a recent IPD of dietary vitamin D requirements in white individuals [23]. Specifically, the present IPD meta-analysis will rely on predefined criteria previously used by the Institute of Medicine (IOM) in their 2011 Dietary Reference Intake (DRI) exercise [2] in terms of selection of RCTs considered most appropriate to address the specific question of setting dietary requirements for vitamin D to meet pre-specified 25(OH)D thresholds. This means in particular that priority is given to the identification of the intake values to maintain serum 25(OH)D concentrations above chosen cut-offs when ultraviolet B (UVB)-induced skin synthesis of vitamin D is absent or markedly diminished. This approach also aligns with that taken by the IOM [2], the Nordic Council of Ministers (NORDEN) [4], and, more recently, the Scientific Advisory Committee on Nutrition (SACN) [14] and the European Food Safety Authority (EFSA) [3].

Within the Population Intervention Comparison Outcome (PICO) framework [26], the populations of interest in this study are specified as dark-skinned male and female children and adults, but excluding studies in infants, toddlers, and pregnant or lactating women as these are life-stage groups that have special considerations in relation to vitamin D [23]. In the present protocol, ‘dark-skinned’ is being defined as those populations with Fitzpatrick skin types of V and VI. However, being aware that Fitzpatrick skin type is not always measured or reported within studies of dark-skinned individuals, should that be the case in identified studies, particular emphasis will be placed on studies which were performed in black individuals, as this is the population group most studied to-date in terms of vitamin D requirements [15–22]. There is a lack of global consensus on racial and ethnicity categories; there are no World Health Organisation- or United Nations Educational, Scientific and Cultural Organisation (UNESCO)-defined terms. Recently, a systematic review of knowledge gaps concerning disease risk in individuals with darker skin has identified the lack of clear definitions in relation to race and ethnicity as a major knowledge gap in the literature [27].

Studies on animals, and patient groups with diseases that are assumed to affect vitamin D metabolism (as outlined in detail elsewhere [26]) and/or response to vitamin D supplementation, will be excluded. The following inclusion and exclusion criteria will be applied:

1. *Intervention*: vitamin D_3_ administered orally on a daily basis—which can be via daily supplements or fortified foods. Inclusion of RCT arms will be limited to a maximum supplemental dose of vitamin D_3_ of 4000 IU (100 μg)/day. Those RCT arms with higher supplemental doses of vitamin D_3_ will be excluded. This selection of upper maximum dose aligns with that used recently by EFSA [3] and takes account of the tolerable upper intake level (UL) for vitamin D of 4000 IU/day for those aged upwards of 9 and 11 years, as set by EFSA [28] and IOM [2], respectively. It also allows for a trend among adults, irrespective of race/ethnicity, for increasing use of higher dose vitamin D supplements [29].
  Inclusion of vitamin D_3_, not vitamin D_2_, on the basis that the IOM DRI committee and EFSA used studies with vitamin D_3_ in their regression analyses, upon which DRI/DRV values were set [2, 3], and, while there is still some debate, there is evidence that the relative potency of vitamin D_2_ in terms of raising serum total 25(OH)D is lower than that achieved with vitamin D_3_ [30, 31]. While the IOM only selected studies which provided vitamin D alone and not with co-administration of calcium [2], both NORDEN [4] and EFSA [3] allowed studies which co-administered calcium to be included. We have provided RCT data to suggest that calcium intake does not influence the response of serum 25(OH)D to vitamin D supplementation [32] and DRVs for vitamin D are established under the assumption that calcium intake is adequate [2–4, 14]. Thus, we are prioritising the approach of NORDEN and EFSA over that of IOM in the present work and will allow RCTs that provided vitamin D alone or co-administered with calcium to be included.
2. RCTs with vitamin D metabolites (25(OH)D and 1,25(OH)_2_D) and analogues (e.g. alfacalcidol) as the intervention will be excluded, as well as RCTs in which the vitamin D was not administered via an oral route. RCTs, which supplied vitamin D in weekly or less frequently (e.g. monthly, quarterly, annually) doses, will also be excluded.
3. *Outcome* and *comparator/comparison:* reported serum or plasma 25(OH)D concentration following supplementation in at least one vitamin D intervention group and one control/placebo group should be available. In a systematic review of existing and potentially novel functional markers of vitamin D status, circulating 25(OH)D has been shown as a robust and reliable marker [33]. In addition, serum 25(OH)D was used as a functional indicator of vitamin D status by authorities briefed with establishing DRV for vitamin D [2–4, 14]. Studies with no data on measured serum or plasma 25(OH)D will be excluded. A conversion factor of 2.496 nmol/L = 1 ng/mL will be used to standardise all circulating concentration to nmol/L.
4. The study duration should be at least 6 weeks as serum 25(OH)D concentrations in adult and elderly subjects only reach equilibrium after 6–8 weeks of vitamin D supplementation [34]. Studies of a duration less than 6 weeks will be excluded.
5. Only studies conducted during, or at least incorporating, winter to ensure minimal impact of UVB on the vitamin D intake**-**25(OH)D dose-response relationship, and thus the calculated vitamin D intake requirements to achieve serum 25(OH)D thresholds, will be included. EFSA in their recent vitamin D DRV analyses defined a period of assumed minimal endogenous vitamin D synthesis at latitudes ≥ 40° N (covering much of Europe) as of October through April [3]. Thus, we will only include data from an RCT if it took place entirely within the window of October and April or has an intermediate sampling point within this winter period and of at least 6 weeks of vitamin D supplementation.
6. Assessment of vitamin D intakes will be based on a food frequency questionnaire, a dietary history, a 24-h recall for ≥ 3 days, or a food record or diary for ≥ 3 days, as per [26]. We will use the total vitamin D intake, which is the total vitamin D intake from the diet as well as that from any supplemental vitamin D dose provided in the RCT [35–37]. The use of total vitamin D intake to derive DRV has been prioritised by expert agencies and bodies [2–4, 14]. Thus, RCTs that have not assessed habitual vitamin D intake in study participants will be excluded.

#### Identification of studies: information sources and search strategy

We will search three online databases (PubMed, Ovid Medline, and Embase) as well as three trial registries (ClinicalTrials.gov, Cochrane Central Register of Controlled Trials (CENTRAL), and the International Standard Randomized Controlled Trials Number (ISRCTN) registry) using electronic search strategies. We will present our full final electronic search strategy, including search strings, using Medline as an exemplar. These searches will be supplemented by searches of review/systematic review articles and reference lists of trial publications as well as from the key international vitamin D DRV/DRI reports over the last 7 years [2–4, 14]. Studies that fulfil the above inclusion criteria and were not already retrieved from the database search will be added.

#### Study selection and inclusion

Study selection will be independently conducted by two pre-specified investigators from among the wider IPD team, first by a screen of the titles and abstracts, followed by a review of the full text of potentially relevant studies. The two investigators will separately determine which RCTs meet the eligibility criteria (no reviewer may assess an RCT on which he or she is listed as a co-author) and be included. Disagreements about study inclusion will be resolved by consensus. Information on the number of records identified, abstracts and full-text articles screened, and articles excluded and included will be provided. All retrieved trials excluded from the review will be given reasons for exclusion as follows: not a RCT; not vitamin D_3_ or unclear; not a daily dose; a dose of supplemental vitamin D_3_ > 4000 IU/d; less than 6 weeks in duration; sampling outside the pre-specified time period; conducted in a location less than 39.9^o^, in infants, toddlers, pregnant or lactating women, or a patient group in which vitamin D metabolism is altered; no circulating 25(OH) D data; and no habitual vitamin D intake data.

### Data collection processes, data items, and IPD integrity

For each eligible RCT, the collaboration will be requested and negotiated with the principal investigator [38]. For willing collaborators, the terms of collaboration will be specified in a data transfer agreement, signed by representatives of the data provider and of the recipients (Cork Centre for Vitamin D and Nutrition Research, University College Cork and Department of Nutrition, Exercise and Sports, University of Copenhagen). Data will be de-identified at source before transfer by email. The IPD request will include the following variables, where available: baseline data for age, sex, racial or ethnic origin, body weight, BMI, serum 25(OH)D, study allocation (vitamin D versus placebo), habitual vitamin D intake, total vitamin D intake, month of baseline and intermediate/endpoint blood sampling, and compliance as well as endpoint (or equivalent) serum 25(OH)D. On receipt, a pre-specified investigator will assess the data integrity by performing internal consistency checks and by attempting to replicate results of the analysis for group mean/median circulating 25(OH)D response to supplemental vitamin D, as published in the RCT report. Study authors will be contacted to provide missing data and to resolve queries arising from these integrity checks. Once queries have been resolved, clean data will be uploaded to the main study database, which will be held in Excel® V15.30 (Microsoft Corporation, USA).

#### Risk of bias assessment for individual studies

An assessment of the risk of bias in the included RCTs will be performed using the Cochrane Collaboration’s tool for assessing the risk of bias [39]. Two pre-specified investigators will independently assess study quality. Discrepancies will be resolved by consensus.

#### Specification of outcomes and effect measures

Serum 25(OH)D concentration will be the sole outcome considered in the IPD meta-analysis. Likewise, total vitamin D intake will be the only predictor considered. The estimated vitamin D intakes (and corresponding 95% confidence intervals) required to keep specified proportions of individuals over a range of pre-specified serum 25(OH)D thresholds will be the effect measures of interest.

### Synthesis and statistical methods

As the primary analysis, a one-stage IPD meta-analysis will be carried out [40, 41]. Previously, both linear and nonlinear dose-response models for describing the relationship between serum 25(OH)D and total vitamin D intake have been used [9, 10, 15–19, 35, 36]. We will consider both types of model as the latter have been suggested to better accommodate the results from RCTs using higher supplemental doses of vitamin D (i.e. up to 2000/4000 IU/day) [2, 3].

Assuming a linear dose-response relationship will imply that the one-stage IPD meta-analysis corresponds to fitting a linear mixed model with vitamin D intake as the fixed effect and study-specific random intercept and slope effects. In contrast, assuming a nonlinear dose-response relationship will lead to a nonlinear mixed-effects model for the one-stage IPD meta-analysis. Specifically, we will assume a three-parameter asymptotic regression model of the form *y* = *b*_2_ + *b*_0_ · [1- exp.(−*x*/*b*_1_)] (with the three parameters *b*_0_, *b*_1_, and *b*_2_), which has been used previously [10], for the fixed-effects part and, if possible, study-specific random effects assigned to all three parameters. Nonlinear mixed-effects models may be more difficult to fit than linear mixed models due to the more complex estimation procedures (Lindstrom-Bates linearization or quadrature methods [42], resulting in a lack of convergence or convergence to sub-optimal parameter estimates. Therefore, we will use two-stage IPD meta-analysis as a sensitivity analysis: It may be less difficult to fit nonlinear dose-response regression models separately per study as it only involves (nonlinear) least squares estimation.

#### Derivation of estimated DRVs

Estimation of required vitamin D intakes to maintain 50, 90, 95, and 97.5% of the participants’ above serum 25(OH)D thresholds of 25, 30, 40, and 50 nmol/L (where feasible) will be achieved by means of inverse regression applied to the predicted mean dose-response curve and the lower boundary of its prediction band. Standard errors and 95% CIs on these derived estimates will be obtained using a bootstrap procedure with 1000 replications, as described previously [9, 23].

#### Additional analyses

To reduce heterogeneity between studies, we will carry a number of additional IPD meta-analyses where additional covariates are included as fixed effects in the mixed model.

##### Body mass index

BMI has been inversely associated with circulating 25(OH)D [43]. Neither IOM nor NORDEN included BMI in their models [2, 4], and while EFSA tested an effect of BMI, it was not included as a covariate in their final model [3]. Recently, the inclusion of BMI as an additional covariate showed minimal alteration in estimates [23]. However, all of these analyses were of predominantly white individuals. Therefore, we will include BMI as an additional covariate in our analysis.

##### Age

The IOM found no evidence of an age effect on the response of serum 25(OH)D to increasing vitamin D intake and therefore included summary data from RCTs in both children and adults within their total dataset [2]. NORDEN likewise combined data from RCTs in children and in adults [4], while EFSA tested the impact of restricting the dataset to only adult RCT arms and excluding those from children. EFSA concluded that the overall estimates did not significantly change compared to the full dataset including children and accordingly retained data on children and on adults in their analyses [3]. It is possible that age, as a surrogate for body size, may impact on the DRV estimates, and in fact, EFSA suggested that children tended to achieve the same mean serum 25(OH)D concentrations as the adults at a lower total intake [3]. Therefore, we will include age as an additional covariate in our analysis.

##### Compliance

Compliance to vitamin D supplements has been reported as being lower in dark-skinned than in white participants in some RCTs [15, 17]. Compliance below 85–95% may affect estimated DRVs. Therefore, additional meta-analyses for different levels of compliance will also be carried out.

#### Statistical software

All analyses will be conducted using R version 3.5.0 (R Core Team, Vienna, Austria) and the R extension packages ‘boot’ for bootstrapping; ‘medrc’, ‘lme4’ [44], and ‘nlme’ [45] for the one-stage IPD meta-analysis; and ‘metafor’ for the two-stage IPD meta-analysis [46]. The Additional file 1 contains R code examples both for linear and nonlinear models.

## Discussion

In light of recent findings showing that dark-skinned population subgroups are at increased risk of vitamin D deficiency [6–8], a more comprehensive investigation of dietary recommendations for vitamin D in these subgroups is much needed. The present study protocol outlines IPD meta-analyses that will allow us to estimate relevant DRVs while utilising all the data that are available on dark-skinned population subgroups.

This will be a collaborative effort involving collaboration with investigators of the eligible RCTs. The present protocol may also facilitate the contact with investigators in terms of gauging how much IPD should be provided and whether the investigators would in theory be willing to share the data.

Finally, beyond its importance in its own right, the protocol will also be of value to a wider audience in terms of highlighting the utility of the IPD meta-analysis for estimating dietary requirement estimates for vitamin D in other at-risk target population subgroups.

## Additional file

Additional file 1: R code examples for the one-stage and two-stage IPD meta-analyses using linear and nonlinear models. (DOCX 14 kb)

## Abbreviations

25(OH)D: 25-Hydroxyvitamin D
DRI: Dietary Reference Intake
DRVs: Dietary Reference Values
EFSA: European Food Safety Authority
IOM: Institute of Medicine
IPD: Individual participant-level data
NORDEN: Nordic Council of Ministers
RCTs: Randomised controlled trials
SACN: Scientific Advisory Committee on Nutrition
