# Supplementary material for: Individual participant data (IPD)-level meta-analysis of randomised controlled trials among dark-skinned populations to estimate the dietary requirement for vitamin D
Source: Syst Rev. 2019 May 28;8:128. doi: 10.1186/s13643-019-1032-6 (PMC6540379; doi:10.1186/s13643-019-1032-6)
Supplement: Supplementary file 1 — R code examples for the one-stage and two-stage IPD meta-analyses using linear and nonlinear models. (DOCX 14 kb) [file 13643_2019_1032_MOESM1_ESM.docx]

**Additional file 1**

The R code snippets for the one-stage and two-stage IPD meta-analyses are shown below.

*Assuming a linear dose-response relationship*

## One-stage IPD meta-analysis

library(lme4)

linear1 <- lmer(serum level ~ intake + (1 + intake | study), data = pooled data)

summary(linear1)

## Two-stage IPD meta-analysis

## Stage 1

linear2.1 <- lm(serum level ~intake, data = study1)

linear2.2 <- lm(serum level ~ intake, data = study2)

…

## Stage 2 (using estimated slopes and their standard errors - from Stage 1)

library(metafor)

linear2 <- rma(estimate, squared standard error, data = aggregate data)

summary(linear2)

*Assuming a nonlinear dose-response relationship (asymptotic regression: AR)*

## One-stage IPD meta-analysis

library(devtools)

install_github("DoseResponse/medrc")

library(medrc) # a wrapper for the package “nlme”

nonlinear1 <- medrm(serum level ~ intake, data = pooled data,

fct = AR.3(), random = b + d + e ~ 1|study)

summary(nonlinear1)

## Two-stage IPD meta-analysis

## Stage 1

nonlinear2.1 <- drm(serum level ~ intake, data = study1, fct = AR.3())

# drm() is in “drc”, which is loaded by “medrc”

nonlinear2.2 <- drm(serum level ~ intake, data = study2, fct = AR.3())

…

## Stage 2 (using vectors of estimated parameters and their standard errors – from Stage 1)

library(metafor)

nonlinear2 <- rma.mv(estimates, squared standard errors,

random = ~1 | study, data = aggregate data)

summary(nonlinear2)
